# Torsion of the spiral colon in cattle– a retrospective analysis of 58 cases

**DOI:** 10.1186/s13028-024-00738-w

**Published:** 2024-04-15

**Authors:** Ueli Braun, Christian Gerspach, Claudia Volz, Monika Hilbe, Karl Nuss

**Affiliations:** 1 Department of Farm Animals, Vetsuisse Faculty, University of Zurich, Zurich, Switzerland; 2 Institute of Veterinary Pathology, Vetsuisse Faculty, University of Zurich, Zurich, Switzerland

**Keywords:** Cattle, Caecum, Ileus, Spiral colon, Torsion

## Abstract

**Background:**

Torsion of the spiral colon (TSC) describes twisting of the spiral colon around its mesentery. The present study reviewed the medical records of 58 cows and heifers with TSC and described the findings, treatment and outcome.

**Results:**

All cases had an abnormal general condition, and the main vital sign abnormalities were tachycardia (72.4%), tachypnoea (67.2%) and decreased rectal temperature (51.8%). Signs of colic were seen in 62.1% of the cows. The most common intestinal abnormalities were an empty or almost empty rectum (96.6%), reduced or absent rumen motility (93.2%), positive ballottement and/or percussion and simultaneous auscultation on the right side of the abdomen (87.9%), reduced or absent intestinal motility (84.5%) and dilatation of the large intestines (spiral colon and/or caecum, 70.7%) diagnosed by transrectal palpation. The main biochemical changes were hypermagnesaemia (70.8%), hypocalcaemia (70.8%), and acidosis (66.7%). Haemoconcentration was found in 63.8%. The main ultrasonographic findings were reduced to absent small intestinal motility (83.3%), dilated small intestines (69.6%) and ascites (66.7%). The spiral colon was dilated in 44.0% of the cows and the caecum in 24.0%. The actual site of torsion could not be visualised. Based on the clinical findings, TSC was diagnosed in 22.4% and caecal dilatation in 50.0% of the cows. A tentative diagnosis of small intestinal ileus was made in another 10.3% of the cows, and a definitive diagnosis of small intestinal ileus in 17.3%. Fifty-three cows underwent right flank laparotomy, and the TSC could be reduced in 26. Twenty-six of the 58 (44.8%) cows were discharged and 32 (55.2%) were euthanased before, during or after surgery.

**Conclusions:**

Acute illness, a sparse amount of faeces in the rectum and dilated spiral colon and caecum are characteristic findings of TSC. The final diagnosis often relies on the surgical or postmortem findings. Cattle with TSC should be treated surgically without delay. The prognosis is guarded with a survival rate of 44.8%.

**Supplementary Information:**

The online version contains supplementary material available at 10.1186/s13028-024-00738-w.

## Background

The colon of cattle consists of the ascending (*colon ascendens*), transverse (*colon transversum*) and descending colon (*colon descendens*). The ascending colon is divided into the proximal, spiral and distal loops [1–3]. The spiral colon is situated directly on the mesentery and consists of centripetal and centrifugal gyri, which form a flat oval construct positioned longitudinally in the abdomen. In a study examining the integration of the spiral colon into the mesentery, 8 intestinal loops were counted from dorsal to ventral in the majority of 213 cases, and 10, 6 or 4 loops were rarely seen [4]. The last (outer) centrifugal gyrus passes into the distal loop of the ascending colon, which continues to the transverse and descending colon and the rectum. The spiral colon itself is rarely affected by ileus but dysmotility can result in obstruction, and extraluminal lesions such as fat necrosis, cysts, neoplasia, haematoma or adhesions can compress it [5]. Case reports have described caecocolic intussusception [6], intussusception of the distal loop of the ascending colon into the spiral colon [7] and spiral colon intussusception [8]. Although rare, torsion of the spiral colon (TSC) describes isolated rotation of the spiral colon around its mesentery [9]. We are aware of only 2 reports, one of which described 3 cattle with an empty rectum, caecal dilatation and taut mesenteric strands detected during transrectal palpation [10]. The other report concerned a 7-month-old calf that had apathy, a dilated abdomen, positive ballottement/percussion and simultaneous auscultation on the right side and taut intestinal structures on transrectal palpation. The principal postmortem finding was torsion of the spiral colon, which was not associated with the mesentery and therefore freely movable [9]. The same study described other cattle with intestinal illnesses and anatomical anomalies of varying extent relating to the structure and position of the spiral colon [9]. The loops of the spiral colon were isolated from the mesojejunum in different configurations, which rendered them more or less freely movable. These changes in the spiral colon, in which the centrifugal and centripetal gyri are arranged atypically and not located adjacent to the left side of the mesojejunum, have been referred to as heterotopy of the spiral colon [11]. The frequency of heterotopy of the spiral colon was investigated in a study of 213 cattle, in which the spiral colon was assessed during laparotomy or postmortem examination [4]. In 60.6% of the cases, the spiral colon was inconspicuous and normally integrated into the mesojejunum. In 24.9%, the heterotopy was minor and termed grade 1, in 9.8% of the cases, large parts of the spiral colon were not integrated into the mesentery (grade 2), while in 4.7%, the spiral colon was completely non-integrated into the mesojejunum (grade 3). The grade-2 anomaly was subsequently termed partial dystopia and the grade-3 anomaly complete ectopy [11]. However, the anomaly was associated with the disease process in only 3 of the 213 cattle: all calves had a grade-3 anomaly. In 2 of the calves (9 and 16 days old), the caecum was rotated around the free loops of the spiral colon, which had a torsion in 1 calf. The third calf (5 weeks old) had an elongated mesojejunum and a severely dilated spiral colon, which was cone-shaped similar to the porcine colon. A study of 1,113 healthy slaughter calves found a frequency of 6.1% for partial dystopia and 2.1% for ectopia of the spiral colon, which was in agreement with the results of previous studies [4, 11, 12]. Genetic factors as well as early manual pregnancy examination between gestation days 32 and 40 are assumed causes of the anomalies [4, 11]. The latter assumption was based on the results of earlier studies of pregnant cattle, which strongly suggested a possible traumatic aetiology of intestinal atresia [13, 14]. Published information relating to TSC in cattle is scarce. The goal of this study was therefore to expand our knowledge by describing the clinical, laboratory and ultrasonographic findings of 58 cattle with TSC and to analyse the course and treatment of this disorder.

## Methods

The medical records of 58 cattle diagnosed with TSC between May 26, 1986, and March 7, 2019, at the Department of Farm Animals, University of Zurich, were analysed (0.002% of the case-load). The present work was based on a dissertation [15]. All cows underwent the same structured clinical examination procedure conducted by the first author (UB) or under the supervision of the first or second author (CG).

### Inclusion and exclusion criteria

Only medical records of cattle that were at least 1 year of age and had TSC at the time of admission were included provided that the diagnosis was confirmed by laparotomy or postmortem examination.

### Clinical examination

All cows underwent a standard clinical examination [16–19], which was based on the examination protocol used at the Clinic of Ruminants, University of Munich, and maintained throughout the study. Each cow was observed for signs of pain. Signs of pain were judged as mild, moderate or severe as described [20]. In addition, the cows were assigned to the colic, indolence or intoxication phase depending on the stage of illness [17, 20].

### Laboratory analyses

The collection and examination of blood, urine and rumen fluid were done as previously described [21].

### Ultrasonographic examination of the abdomen

The abdomen of 25 more recent cases was scanned from the right side as described [22]. The first or second author was present during the ultrasonographic examinations except for cows admitted at night, in which case the ultrasonographic images were evaluated the following morning. The pictures were saved and re-evaluated for this study.

### Diagnosis

A tentative clinical diagnosis of small or large intestinal ileus was made in cattle with a history of colic or with signs of colic at the initial examination and with no or only a sparse amount of faeces in the rectum. A tentative diagnosis of small intestinal ileus was made when dilated small intestines were palpated during transrectal examination. A diagnosis of caecal dilatation (CD) was made when the caecum was dilated and displaced caudally [23]. The clinical diagnosis of TSC was made during transrectal examination when the spiral colon was severely dilated and had a “corrugated-iron-like” pattern. The latter term describes dilated, tense, full loops of spiral colon that are concentrically arranged; the term has been used to describe transrectal findings in cows with mesenteric torsion [18, 33]. Often, this was accompanied by CD, and the cattle had a moderately to severely abnormal demeanour. In cattle with dilated small and large intestines and a severely abnormal general condition, a diagnosis of ileus attributable to mesenteric torsion (MT) was made [18]. An ultrasonographic diagnosis of ileus was based on the identification of dilated small intestines with a diameter ≥ 4.0 cm and greatly reduced or absent motility in cattle with clinical signs suggesting ileus. Involvement of the large intestine in the ileus was suspected when gas- or fluid-filled segments of the large intestine were seen [24] and the wall of the dilated large intestine closest to the transducer could be imaged as a thick echogenic line. The caecum and the proximal loop of the colon appeared as thick, echogenic, continuous and slightly curved lines, whereas the spiral loop of the colon had the appearance of a garland with several echogenic arched lines next to each other. The gold standard for diagnosis was laparotomy findings in operated cattle and/or postmortem findings in cattle that were euthanased. A surgical and/or postmortem diagnosis of TSC was made in cattle with an isolated torsion of the spiral colon around its mesentery, possibly involving the caecum and/or parts of the small intestine, detected during surgery or postmortem examination.

### Laparotomy

Right-flank laparotomy was carried out in standing cattle or in left lateral recumbency [18]. Cattle that had difficulty standing because of severe illness or visceral pain were operated in left lateral recumbency. Distal paravertebral analgesia was mainly used until 2001, after which time proximal paravertebral analgesia was used. A 25- to 30-cm incision was made in the mid-paralumbar fossa, which allowed initial visual assessment of the intestines. Markedly gas-filled sections of large intestine were deflated using a hypodermic needle attached to rubber tubing, and the centesis site was closed using a cruciate suture pattern. First, the dilated caecum was exteriorised according to [23]. An incision approximately 4 to 5 cm in length was made in the apex of the caecum and the contents were emptied into a bucket. The remainder of the caecum, the proximal loop of the ascending colon and, if possible, the obstipated parts of the spiral colon were also emptied via gentle massage. The caecal incision was closed using monofilament absorbable suture material in a double-layer Cushing suture pattern, and the enterotomy site was flushed with diluted iodine solution. When the caecum rapidly re-filled with ingesta, it was incised and emptied a second time. When there was absence of caecal contractions, caecal necrosis or recurrence of the dilatation following a previous surgery, amputation of the caecum was carried out close to the caeco-ileal junction [23]. The caecal stump was closed using resorbable suture material in a double-layer Cushing suture pattern, and the site was flushed with iodine-saline solution. The next step was to determine the direction of the TSC and to reduce the torsion. The abdomen was thereafter closed in four layers using resorbable suture material, and metal clips were used to close the skin.

### Postoperative treatment

The cows that were successfully operated and subsequently discharged were fasted for at least 24 h postoperatively before feeding was gradually resumed. They received fluid therapy, antibiotics, analgesics, prokinetic drugs and electrolyte replacement.

### Euthanasia/slaughter

Cattle were euthanased using pentobarbital during or after the initial examination when they were in the intoxication phase or when the owner did not consent to surgery. In earlier years, they were sent to the slaughter facility of the Veterinary Hospital and the meat was used for zoo-animal feeding. Cattle were euthanased intraoperatively when lesions associated with a very poor prognosis (e.g., ruptured intestines, fibrinous peritonitis, haemorrhagic infarction) were seen or complications (e.g., becoming recumbent on the right side during surgery with exteriorization and subsequent contamination of the intestines) occurred, and postoperatively when the clinical condition deteriorated.

### Postmortem examination

All cattle that died or were euthanased underwent postmortem examination. In slaughtered cattle, only the internal organs were inspected.

### Statistical analysis

The program SPSS Statistics 26.0 (IBM Corp. 2017, USA) was used for analysis. Frequencies were determined for all variables, and the Shapiro-Wilk test was used to test the data for normality. Means ± standard deviations were calculated for normal data and medians (with range) for non-normal data. In addition, the 95% confidence intervals (CI) were calculated for the means and medians. Differences in non-normal data between surviving (from admission to hospital discharge) and non-surviving cows were analysed using the Mann-Whitney U test, and differences in nominal data were analysed using the chi-square test (*n* ≥ 5) or two-tailed Fisher’s exact test (*n* < 5). A value of *P* < 0.05 was considered significant.

## Results

### Cattle and history

There were 53 cows and 5 heifers (referred to as cows in the study) ranging in age from 1 to 9 years (median, 4 years). Breeds included Brown Swiss (*n* = 30), Swiss Fleckvieh (*n* = 21) and Holstein (*n* = 7). Of the 58 cows, 34.5% were pregnant, 37.9% were not pregnant and in the remaining 27.6%, the pregnancy status had not been recorded. The duration of pregnancy ranged from 4 to 40 weeks (median, 95% CI, 24.5, 16–34 weeks). The latest calving date was known for 26 cows and was 1 day to 18 weeks before admission (median, 95% CI, 5.2, 2.7–10.0 weeks). The duration of illness before admission ranged from 2 to 14 days (median, 95% CI, 24, 12–24 h). 69.6% (39/56) of the cows had a history of anorexia and 30.4% had a reduced feed intake. 74.1% (43/58) of the cows had a history of colic and 65.5% had received medical treatment before admission.

### General condition, abdominal contour and signs of pain

The general condition was mildly abnormal in 15.5% of the 58 cows, moderately abnormal in 37.9% and severely abnormal in 46.6%. Four cows were recumbent on admission and one other became recumbent during the physical examination. Unilateral or bilateral abdominal distension was seen in 43.1% of the cows. Non-specific signs of pain including bruxism (8.6%), muscle fasciculations (8.6%), spontaneous grunting (3.4%) and piloerection (1.7%) occurred in 22.4% of the 58 cows. Thirty-six were in the colic phase but a detailed description was available in only 33. Signs included treading (27.6%), a sunken back (25.9%), kicking at the belly (24.1%), restlessness (20.7%), frequent lying down and rising (15.5%) and sweating (3.4%) (Additional file 1). A single clinical sign of colic was seen in 24.1% of cows, two signs in 12.1%, three signs in 19.0% and four signs in 1.7%. Signs of visceral pain were mild (19.0%), moderate (15.5%) or severe (29.3%). By the time of admission, 31.0% of the 58 cows had advanced to the indolence phase, characterized by apathy and a markedly abnormal general condition, and 6.9% to the intoxication phase, in which cattle had tachycardia, congested scleral blood vessels, pale mucous membranes, cool skin surface temperature, sunken eyes and/or a dry muzzle. In addition, 73.7% of 57 cows had a tense abdominal wall.

### Heart and respiratory rates and rectal temperature

Of the vital signs, the most common abnormalities were tachycardia (72.4%), tachypnoea (67.2%) and lower-than-normal rectal temperature (51.7%) (Table 1).

**Table 1:** Clinical findings in cows with torsion of the spiral colon (means ± sd, medians, 95% CI, frequency distributions)

Variable	Finding		Number of cattle	%
Heart rate	Normal (60–80 bpm)		16	27.6
(*n* = 58, median= 100 bpm, 95% CI = 88–112 bmp)	Mildly increased (81–100 bpm)		17	29.3
	Moderately increased (101–120 bpm) Severely increased (121–176 bpm)		12 13	20.7 22.4
Rectal temperature	Normal (38.5–39.0 °C)		14	24.1
(*n* = 58, median= 38.4 °C, 95% CI = 38.3–38.8 °C)	Decreased (35.7–38.4 °C)		30	51.7
	Increased (39.1–41.3 °C)		14	24.1
Respiratory rate	Normal (15–25 breaths per min.)		19	32.8
(*n* = 58, median = 28 breaths per min., 95% CI = 28–32 breaths per min.)Must be uncoulored	Increased (26–84 breaths per min.)		39	67.2
Rumen motility (*n* = 58)	Normal Decreased Absent		4 27 27	6.8 46.6 46.6
Foreign body tests (*n* = 50)	All negative At least one test positive^1^		37 13	74.0 26.0
BSA and PSA on the left side (*n* = 57)	Both tests negative (normal) Only BSA positive Only PSA positive		55 1 1	96.4 1.8 1.8
BSA and PSA on the right side (*n* = 58)	Both tests negative (normal) Only BSA positive Only PSA positive Both tests positive		7 14 6 31	12.1 24.1 10.3 53.5
Intestinal motility (*n* = 58)	Normal Decreased Absent		9 27 22	15.5 46.6 37.9
Rectal findings (*n* = 58)	Normal findings Dilated loops of small intestines Dilatation of the caecum Dilatation and retroflexion of the caecum Dilatation and torsion of the caecum Dilatation of the spiral colon Unclear findings		9 5 11 5 4 21 3	15.5 8.6 19.0 8.6 6.9 36.2 5.2
Faeces, amount (*n* = 58)	Normal Faecal output reduced Rectum empty		2 29 27	3.4 50.0 46.6
Faeces, degree of comminution (*n* = 58)	Normal (well digested) Moderately digested Poorly digested Rectum empty		28 1 2 27	48.3 1.7 3.4 46.6
Faeces, consistency (*n* = 58)	Normal Thick pulpy Dry balls Thin pulpy Liquid Biphasic Rectum empty		15 9 2 3 1 1 27	25.9 15.5 3.4 5.2 1.7 1.7 46.6
Faeces, colour and abnormal contents in the rectum (*n* = 58)	Normal (olive) Dark Blood Mucus Fibrin More than one abnormality		21 8 14 6 3 6	36.3 13.8 24.1 10.3 5.2 10.3

^1^ Positive: at least 3 of 4 attempts elicited a grunt

### Digestive tract abnormalities

**Fig. 1:**
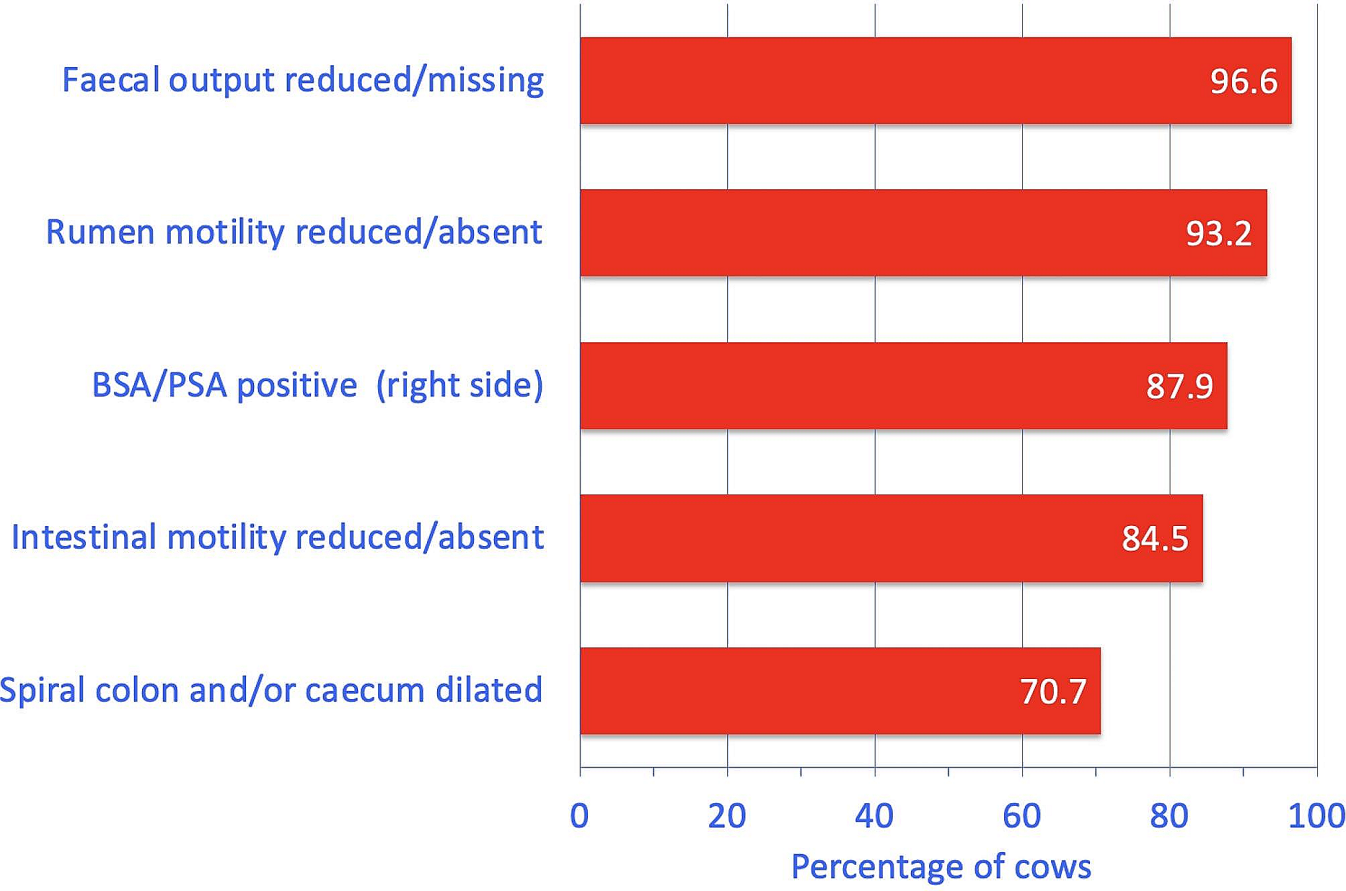
The most common digestive tract abnormalities in 58 cows with torsion of the spiral colon

The most common findings were no or a sparse amount of faeces in the rectum (96.6% of 58 cows), reduced or absent rumen motility (93.2%), positive ballottement and/or percussion and simultaneous auscultation on the right side (87.9%), reduced or absent small intestinal motility (84.5%) and dilated large intestines (spiral colon and/or caecum; 70.7%) on transrectal palpation (Fig. 1; Table 1). Dilated small intestines were detected in only 8.6% of the cows. Other findings were rumen tympany in 15.8% of 57 cows and at least one positive grunt test (pole test, pinching of the withers, and percussion of the abdominal wall over the region of the reticulum with a rubber hammer) in 26.0% of 50 cows. The faeces were dark to black in 13.8% of the 58 cases and the consistency ranged from liquid to pulpy (normal) to thick pulpy. Abnormal faecal contents included blood and fibrin and combinations thereof.

### Other clinical findings

Other clinical findings were reduced skin surface temperature (77.6%), reduced skin elasticity (50.9% of 57 cows), prolonged capillary refill time (49.1% of 55 cows), moderately to severely hyperaemic scleral vessels (45.6%, 26/57), sunken eyes (39.7%, 23/58), a dry and cool muzzle (32.8%, 19/58), pale oral mucosa (23.2%, 13/56) and ammonia-like or otherwise foul breath (17.2%, 10/58).

### Urinalysis

The urine pH of 45 cases ranged from 5.0 to 9.0 (median, 7.0); it was decreased (< 7.0) in 44.4% and increased (> 8.0) in 17.8% of the cows. The specific gravity of 46 samples ranged from 1.002 to 1.055 (mean ± sd, 1.031 ± 13); it was lower than normal (< 1.020) in 19.6% and higher than normal (> 1.040) in another 19.6%. Examination of 48 urine samples using Combur^9^ test strips (Roche Diagnostics) yielded glucosuria (50 to 1000 mg/dL urine) in 33.3%, haemoglobinuria/haematuria (5 to 250 erythrocytes/ µL urine) in 22.9%, ketonuria (10 to 150 mg/dL urine) in 4.2% and proteinuria (100 to 500 mg protein/dL urine) in 2.1% of the cows.

### Laboratory findings

**Fig. 2:**
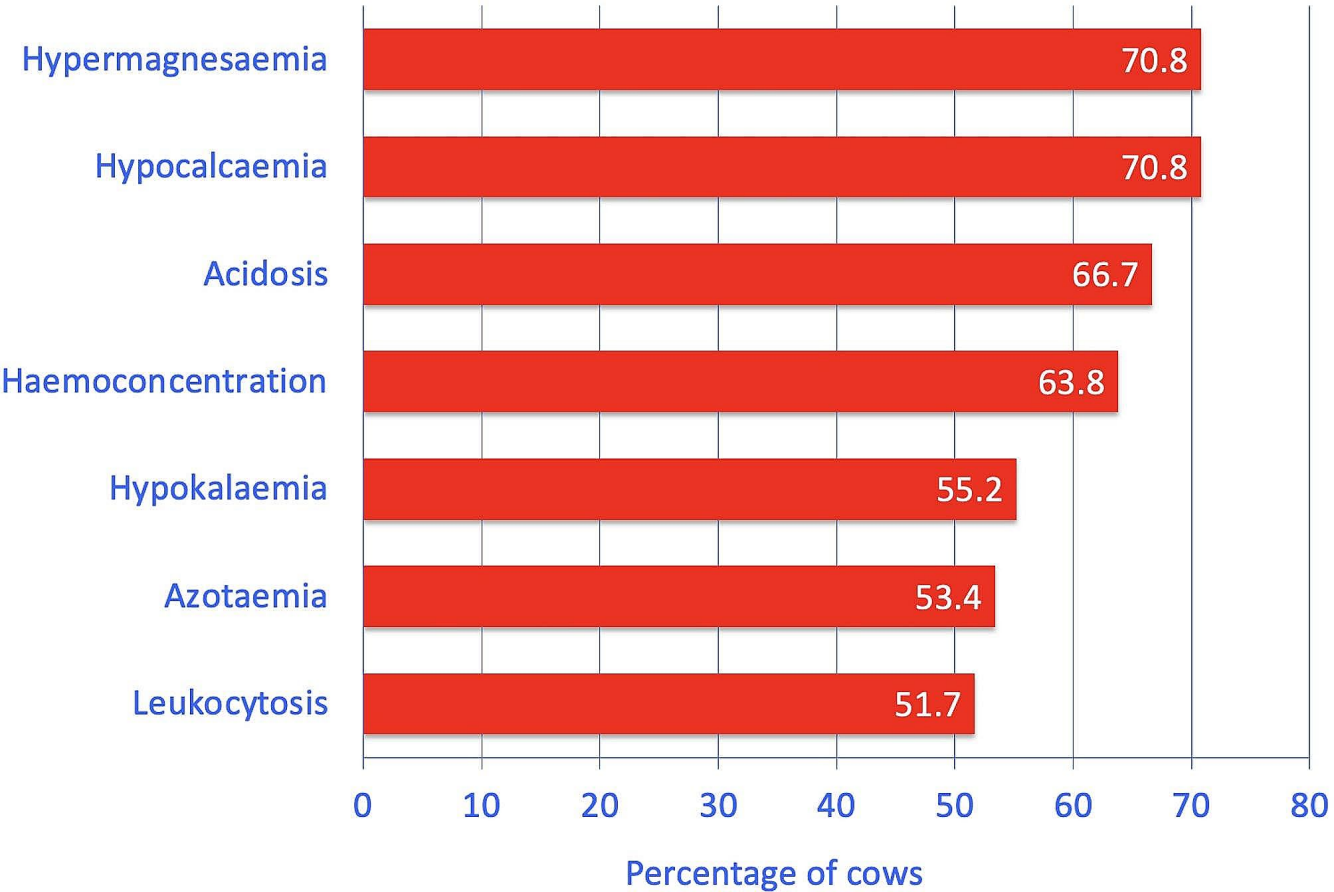
The most common abnormal blood variables in 58 cows with torsion of the spiral colon

The main abnormalities were hypermagnesaemia (70.8%, 17/24), hypocalcaemia (70.8%, 17/24), acidosis (based on blood pH level, 66.7%, 26/39), haemoconcentration (63.8%, 37/58), hypokalaemia (55.2%, 32/58), azotaemia (53.4%, 31/58) and leukocytosis (51.7%, 30/58) (Fig. 2, Additional file 2). Less frequent findings were increased base excess (46.2%, 18/39), increased activity of aspartate aminotransferase (41.4%, 24/58), hypercapnia (34.2%, 13/38), hyperproteinaemia (33.3%, 19/57) and hypophosphataemia (33.3%, 8/24). The rumen chloride concentration was increased in 9.1% (4/44) of the cases.

### Ultrasonographic findings

The principal ultrasonographic findings were analogous to those in cows with small [22] and large intestinal ileus [24]. The spiral colon was dilated in 44.0% (11/25) and the caecum in 24% (6/25) of the scanned cows (Table 2). The small intestinal motility was subjectively reduced or absent in 83.3% (15/18) and the small intestines were dilated in 69.6% (16/23) of the cows. Abdominal fluid was seen in 66.7% (16/24) of scanned cows. Based on the ultrasonographic findings, the location of the ileus was suspected to be the caecum or the spiral colon in 52.0% (13/25), the small and large intestines in 20.0% (5/25) and the small intestines alone in 16.0% (4/25) of the scanned cows. The actual site of the torsion could not be visualised in any of the cases.

**Table 2:** Ultrasonographic findings in cows with torsion of the spiral colon

Variable	Finding		Number of cattle	%
Spiral colon (*n* = 25)	Not evaluated Dilated		14 11	56.0 44.0
Caecum (*n* = 25)	Not assessed Dilated		19 6	76.0 24.0
Cross-section of small intestines (*n* = 23)	Normal (< 4.0 cm) Dilated (4.0–8.4 cm) **√**		7 16	30.4 69.6
Intestinal motility (*n* = 18)	Normal Decreased Absent		3 3 12	16.7 16.7 66.6
Free fluid in the abdomen (*n* = 24)	No fluid visible Fluid without fibrin Fluid with fibrin		8 11 5	33.3 45.9 20.8
Ultrasonographic localisation (*n* = 25)	Caecum Spiral colon Small and large intestine Small intestine No information		10 3 5 4 3	40.0 12.0 20.0 16.0 12.0

### Comorbidities

One to three comorbidities, unrelated to TSC, were diagnosed in 20.7% of the 58 cows including parasitism (gastrointestinal nematodes, dicrocoeliosis, ectoparasites), claw diseases, respiratory problems, ketonuria, udder cleft dermatitis, mastitis and metritis.

### Diagnoses

Based on the results of clinical examination, caecal dilatation was diagnosed in 50.0% of the 58 cows and TSC in 22.4% (Table 3). A tentative diagnosis of small intestinal ileus was made in 10.3% and a definitive diagnosis of small intestinal ileus in 17.3%. Based on ultrasonographic examination of 25 cows, caecal dilatation was diagnosed in 11 (44.0%), TSC in 5 (20.0%), small intestinal ileus in 7 (28%) and mesenteric torsion in 1. An ultrasonographic diagnosis could not be made in the remaining cow.

**Table 3:** Diagnosis of 58 cattle with torsion of the spiral colon

Diagnosis	Clinical diagnosis (*n* = 58)	Ultrasonographic diagnosis (*n* = 25)
Diagnosis of dilatation of the caecum	50.0% (*n* = 29)	44.0% (*n* = 11)
Diagnosis of torsion of the spiral colon	22.4% (*n* = 13)	20.0% (*n* = 5)
Tentative diagnosis of small intestinal ileus	10.3% (*n* = 6)	-
Diagnosis of small intestinal ileus	17.3% (*n* = 10)	28.0% (*n* = 7)
Diagnosis of mesenteric torsion	-	4.0% (*n* = 1)
No diagnosis	-	4.0% (*n* = 1)

### Treatment and outcome

**Fig. 3:**
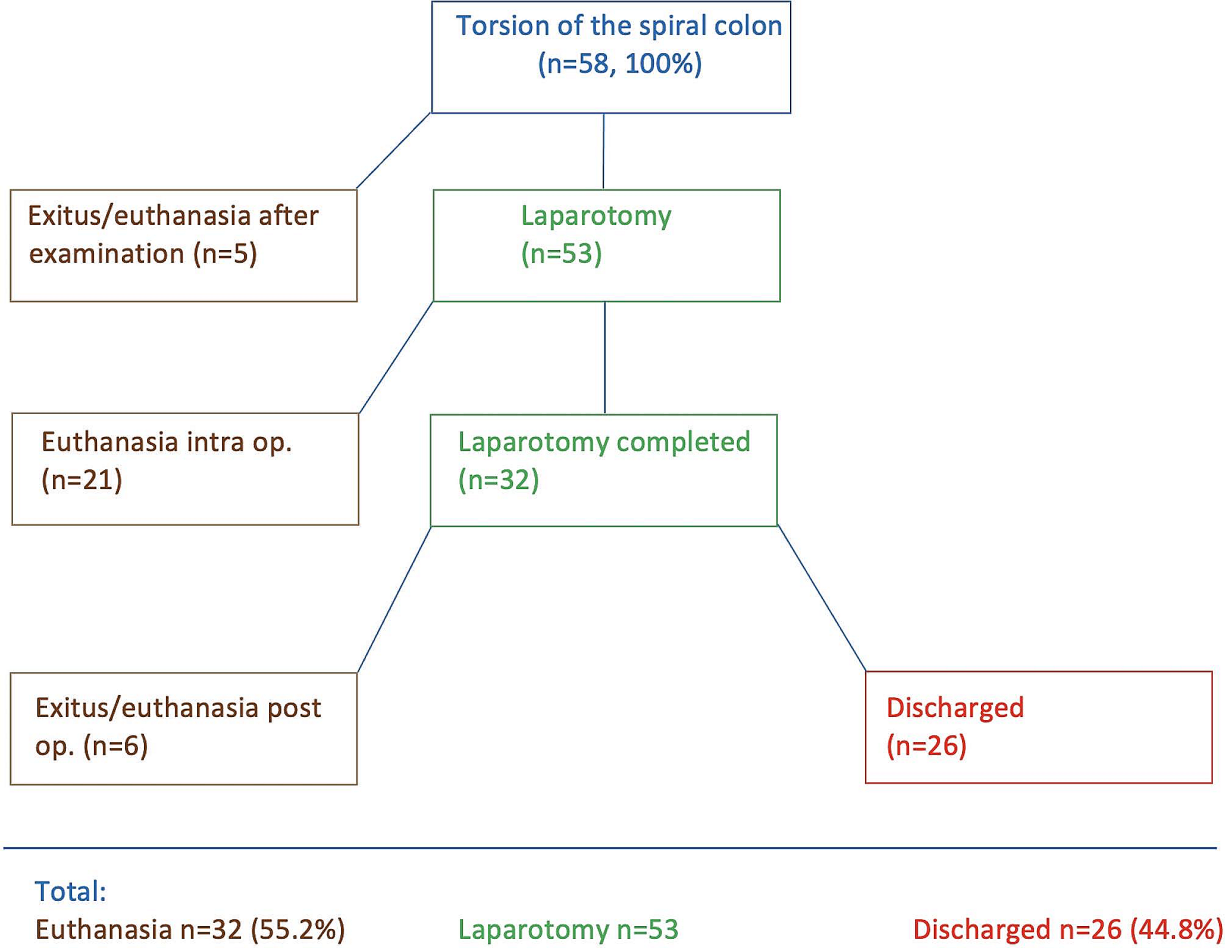
Treatment flowchart for 58 cattle with torsion of the spiral colon

Five cows died or were euthanased during or shortly after the initial examination because they were in the intoxication phase or the owner did not consent to surgery (Fig. 3). Of the 58 cows, 91.4% underwent right flank laparotomy and 39.6% of these were euthanased intraoperatively because of lesions associated with a very poor prognosis or complications. The surgery was completed in 32 cows, 6 of which were euthanased postoperatively. Twenty-six cows underwent postoperative medical treatment and were discharged. In summary, 32 of 58 (55.2%) cows died or were euthanased and 26 (44.8%) were discharged.

### Surgical findings and intraoperative complications

Fifty-three cows underwent right-flank laparotomy; 46 were operated in a standing position and 5 were sedated and recumbent. In 2 cases, the operation was started with the cows standing and finished with the cows in sternal or lateral recumbency because the cows became recumbent. Of all these 53 cows with TSC, the torsion was limited to the spiral colon in 47.2% of the cows, the spiral colon and the caecum in 43.4%, and in the remaining 9.4% of the operated cows, the small intestine was involved in the torsion in addition to the spiral colon and caecum. 86.8% of the 53 cows also had caecal dilatation, torsion or retroflexion. The caecum was normal in 2 cows. Other common surgical findings were ascites in 39.6% of the 53 cows and secondary small intestinal dilatation in 37.7%. Surgery was completed in 32 cows: a complete TSC (360 degrees) was reduced in 16 and a partial TSC in the remaining 16. In 87.5% (28/32) of these cows, the caecum was evacuated and/or amputated. Euthanasia was carried out intraoperatively in 21 cows because of severe changes or because reduction of the TSC was not possible.

Intraoperative complications occurred in 24.5% of the 53 operated cows and included unexpected recumbency (*n* = 6), tearing of the serosa or mesentery upon manipulation (*n* = 3), death, severe haemorrhage, suboptimal amputation and a combination thereof (each *n* = 1).

### Postoperative treatment

The 26 cows that were operated successfully and subsequently discharged were fasted for at least 24 h postoperatively before feeding was gradually resumed. Medical treatment included fluid therapy, antibiotics, pain control in all animals and prokinetic drugs in 22 and electrolyte replacement in 24 animals.

All cattle received 10 L of a solution containing 50 g glucose and 9 g NaCl/L daily for 1 to 7 days (median, 3 days) administered as a slow intravenous drip via an indwelling jugular vein catheter (Abbocath-T 14 g, length 14 cm, Abbott AG, Baar). Antibiotic treatment included penicillin G procaine (12,000 IU/kg) given intramuscularly (18/26), amoxicillin (7 mg/kg) given intramuscularly (3/26), penicillin G procaine and amoxicillin combined (4/26) and sulfadoxine-trimethoprim (15 mg/kg) given intravenously (1/26). The antibiotic treatment was in most cases administered for 4 or 5 days (median, 4 days). All cattle received a daily injection of flunixin meglumine (1 mg/kg) or ketoprofen (3 mg/kg), and 12 cows also received metamizole (35 mg/kg) given intravenously for 2 to 5 days (median, 3 days). Prokinetic drugs were used in 22 cattle for a duration of 1 to 7 days (median, 2 days). Twenty-two cattle received neostigmine (40–45 mg) administered via continuous drip infusion and 3 of them also received intramuscular metoclopramide (30 mg), every 8 h, for a total of 7 to 9 treatments (metoclopramide was only used in the first few years). Twelve cows with hypocalcaemia (calcium < 2.0 mmol/L) received 500 mL of 40% calcium borogluconate, administered intravenously. Nine cows with hypokalaemia (K^+^<4.0 mmol/L) were treated with daily oral doses of 60 to 100 g of KCl until normokalaemia occurred. Three cows with hypophosphataemia (inorganic phosphorus < 1.0 mmol/L) were treated orally with sodium dihydrogen phosphate.

### Short-term outcome of the 32 operated cows

Six cows deteriorated and died or were euthanased within 3 days of surgery. The general condition of the remaining 26 discharged cows normalised within 2 to 11 days (median, 4.0 days), feed intake within 3 to 11 days (median, 3.0 days) and faecal output within 1 to 8 days (median, 3.0 days) after surgery. The median rectal temperature ranged from 38.5 to 38.8 °C on days 0 (day of surgery) to 7 postoperatively. The median heart rate, which was mildly elevated at 82 bpm on admission, was in the normal range at 76 bpm one day after surgery and ranged from 72 to 80 bpm in the following days. Seventeen cows were in good health and discharged within 3 to 5 days, 8 cows within 5 to 13 days and 1 cow 34 days after surgery.

### Comparison of the 32 non-surviving and 26 surviving cows

**Fig. 4:**
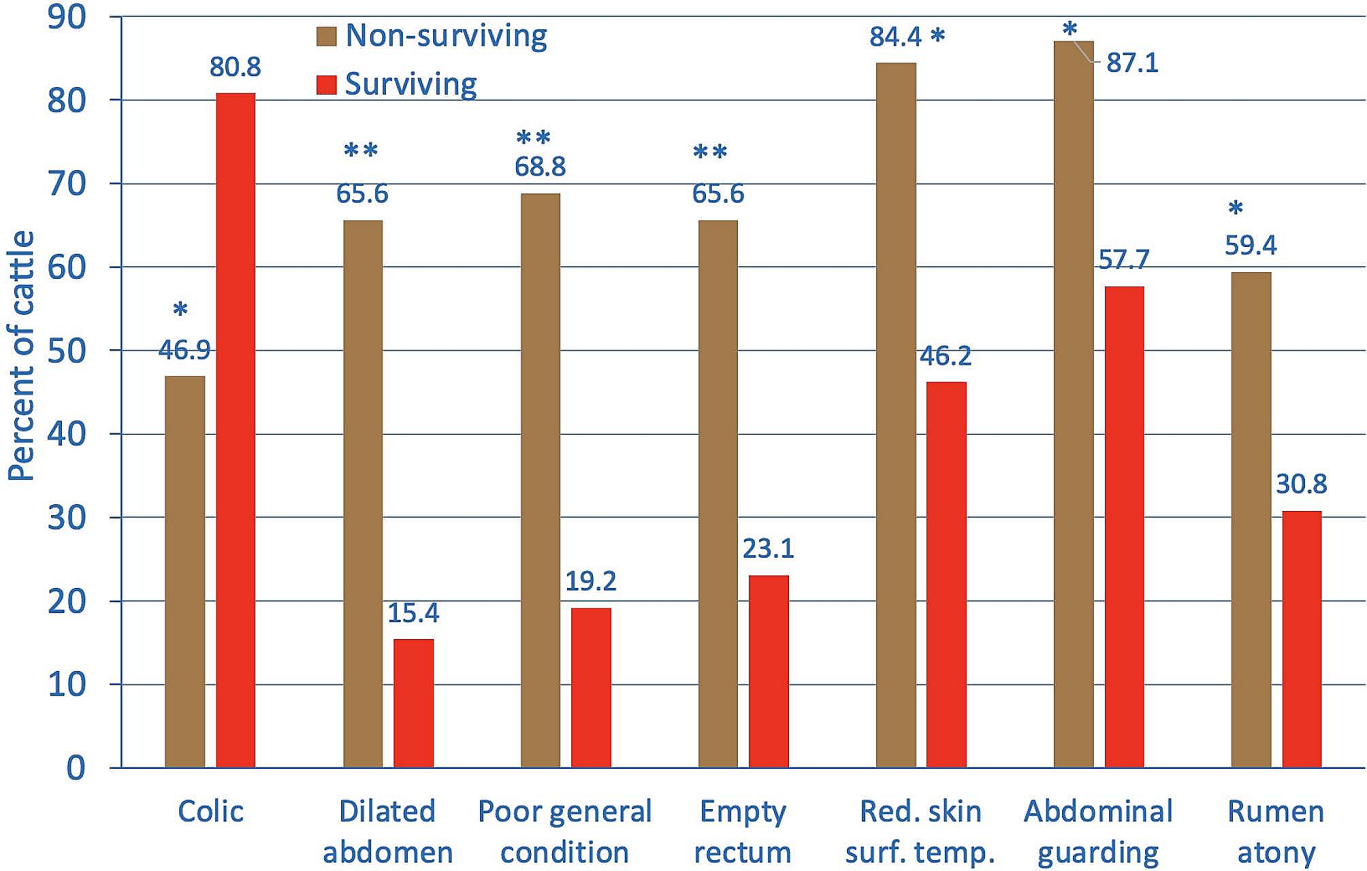
Differences in the frequencies of clinical findings between non-surviving (*n* = 32) and surviving (*n* = 26) cows with TSC. * *P* < 0.05, ** *P* < 0.01

The median heart rate on admission was significantly higher in the non-surviving cows (118 bpm) compared with the surviving cows (82 bpm, *P* < 0.01) and the rectal temperature was significantly lower (38.3 vs. 38.9 °C, *P* < 0.01). Several other clinical variables also differed significantly between the two groups (Fig. 4) but colic was the only variable that occurred more frequently in surviving cows. Non-surviving and surviving cows differed the most with respect to distended abdomen (65.6 vs. 15.4%), followed by severely abnormal general condition (68.8 vs. 19.2%), empty rectum (65.6 vs. 23.1%), reduced skin surface temperature (84.4 vs. 46.2%), tense abdomen (87.1 vs. 57.7%) and rumen atony (59.4 vs. 30.8%). None of the 9 cows with a dilated abdomen, severely abnormal general condition and empty rectum survived.

Surviving and non-surviving cows differed significantly with respect to median haematocrit (35.5 vs. 39.0%, *P* < 0.01), urea concentration (6.0 vs. 8.2 mmol/l, *P* < 0.05) and blood pH (7.41 vs. 7.34, *P* < 0.05).

### Long-term outcome of the 26 discharged cows

The long-term outcome was determined 2 years after discharge via telephone interview. Of the discharged cattle, 10 (38.5%) had remained in the herd and were still productive. TSC had re-occurred in 4 cows, and 5 others were euthanased or slaughtered for economic or other reasons. The long-term outcome was not known for the remaining 7 cows.

### Postmortem findings

**Fig. 5:**
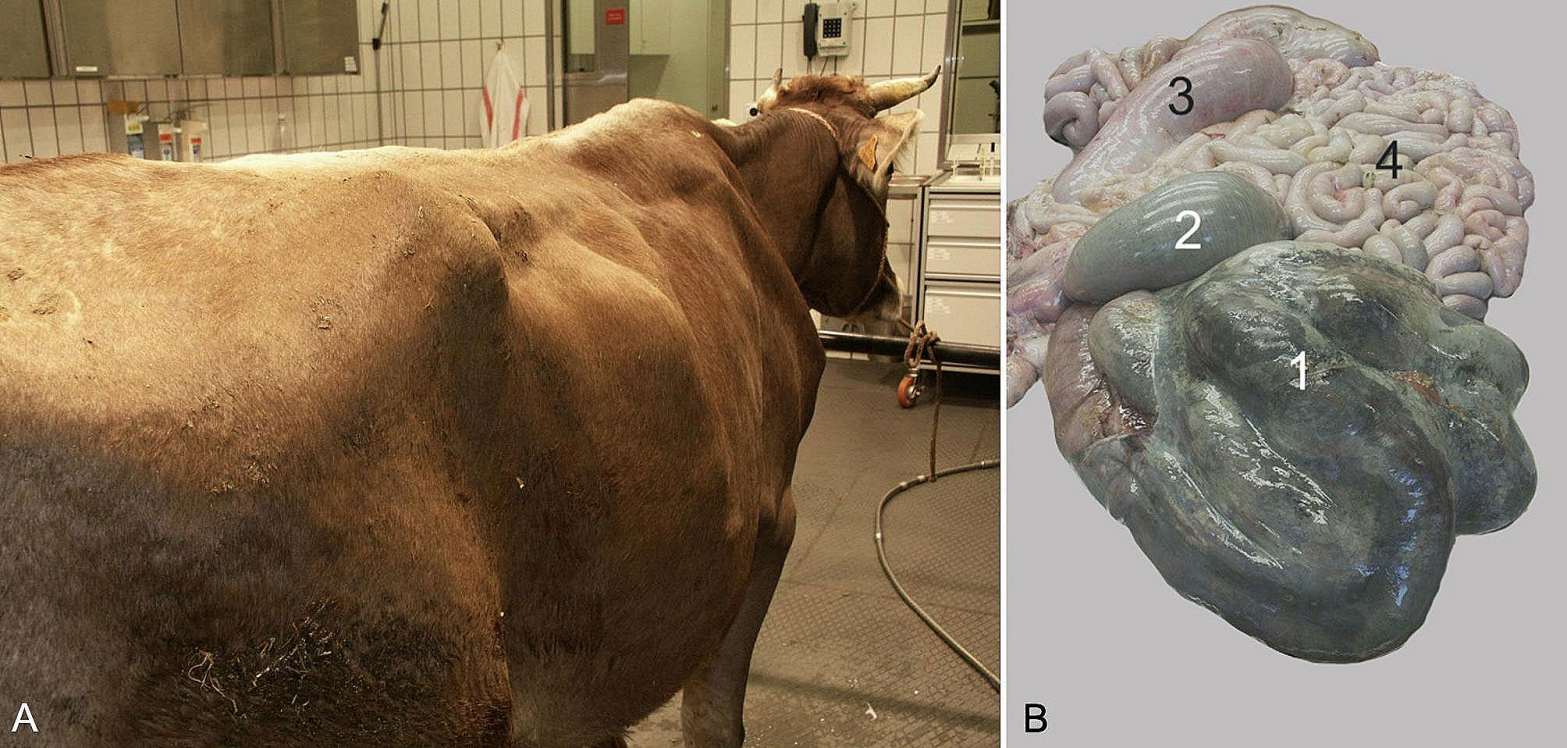
A large bulge in the right flank (**A**) and postmortem findings (**B**) in a 4-year-old Brown Swiss cow with torsion of the spiral colon (also see video). The gyri of the spiral colon (1) and the proximal loop of the ascending colon (2) are congested and severely dilated and have a dark bluish-green discolouration. The caecum (3) is dilated but otherwise normal. The small intestines are mildly to moderately congested but otherwise normal

The main finding in the 32 cows that died or were euthanased was haemorrhage and/or necrosis at the site of torsion at the base of the spiral colon (Fig. 5), often accompanied by dilatation of the caecum and spiral colon, and secondary small intestinal dilatation because of congestion. Five cows had mesenteric haemorrhage and/or oedema, and five others had acute fibrinous peritonitis; the latter was attributable to caecal rupture in one, type-4 abomasal ulcer in one and intestinal necrosis in three cows.

## Discussion

The cause and clinical manifestation of ileus vary in cattle. The clinical signs depend on the location of the ileus and the associated intestinal and mesenteric lesions. Ileus of the duodenum or cranial jejunum rapidly leads to abomasal reflux syndrome and hypochloraemic hypokalaemic metabolic alkalosis [25], whereas the clinical signs of small intestinal intussusception [17] or ileal impaction [26] tend to be mild and characterised by a protracted course. In contrast, the clinical course is much more acute and dramatic in cattle with intestinal torsion [27–31]. The most severe clinical abnormalities are seen in cattle with mesenteric torsion [32–37] because the entire intestinal tract is twisted around the mesenteric root. The severity of illness is greater in cows with TSC than in cows with small intestinal volvulus but less than in cows with mesenteric torsion; a moderately to severely abnormal general condition was seen in 84.5% of cows with TSC compared with 76.6% of cows with small intestinal volvulus and 96.7% of cows with mesenteric torsion. Likewise, 72.4% of cows with TSC had tachycardia, which was similar to cows with small intestinal volvulus (68.0%) [38] but lower than in cows with mesenteric torsion (80.4%) [18] and considerably higher than in cows with small intestinal intussusception (38.1%) [17] and small intestinal strangulation (36.6%) [19]. The frequent occurrence of tachycardia in cows with TSC, small intestinal volvulus and mesenteric torsion is related to the strong haemodynamic effect of intestinal torsion and the rapid onset of toxaemia [37]. An empty or almost empty rectum, on the other hand, is a common clinical finding in cows with mechanical ileus [39], and the frequency in cows with TSC (96.6%) differed little from that of other forms of ileus. Ballottement and/or percussion and simultaneous auscultation were positive on the right side in 87.9% of cows with TSC, which was similar to cows with mesenteric torsion (91.7%) but higher than in cows with caecal dilatation (82.6%) [23], small intestinal volvulus (78.7%) [38], small intestinal intussusception (65.9%) [17], small intestinal strangulation (58.3%) [19] and duodenal ileus (56.5%) [40]. Positive findings are based on gas and fluid accumulations in the small intestine in cows with small intestinal ileus, and primarily on gas and fluid accumulation in the caecum and spiral colon in cows with caecal torsion and TSC. In our experience, it is not possible to reliably differentiate tympanic resonance sounds (“pings”) that originate from the small and large intestines, notwithstanding reports on the relationship between the localisation and the underlying cause of a ping sound [41–43]. At least in cows with mesenteric torsion, it can be assumed that both the small and large intestines are involved in the generation of the ping sound. Dilated small intestines detected during transrectal examination are a tell-tale sign of small intestinal ileus [39], whereas the dilated caecum is prominent in cows with caecal dilatation [44]. Dilated small intestines were rarely detected in cows with TSC (8.6%), whereas dilated large intestines were common (caecum and/or spiral colon 70.7%). The dilated caecum could be palpated transrectally in 87.9% of cows with caecal dilatation, which was considerably more common than in cows with TSC, whereas dilatation of the small intestines (0.9%) or the spiral colon (0.4%) was rare. Dilated large intestines were not palpated in cows with small intestinal intussusception [17] and only rarely in cows with small intestinal volvulus (6.4%) [38], small intestinal strangulation (6.7%) [19] and ileal impaction (13.6%) [26]. In contrast, dilated large intestines (44.1%) together with dilated small intestines (49.2%) are the hallmark transrectal findings in cows with mesenteric torsion [18]. Thus, diagnostically important differences in transrectal findings occur among intestinal disorders characterised by ileus. Summarising, dilated large intestines and the absence of palpable small intestines are typical transrectal findings in cows with TSC.

The clinical findings allowed some conclusions as to the likelihood of survival or non-survival. Cows with bilateral abdominal distension (15.4 vs. 65.6%), a severely abnormal general condition (19.2 vs. 68.8%) and an empty rectum (23.1 vs. 65.6%) had a poor prognosis and none of the 9 cows with these 3 findings survived. Colic at the time of admission was significantly more common in surviving cows (80.8 vs. 46.9%). The most likely explanation for this is that the non-surviving cows were at a more advanced stage of illness, such as the indolence or intoxication phase. The colic phase is short in cattle and only lasts about 12 h [20].

Although ileus results in fluid secretion into the intestinal lumen, we were surprised that the ultrasonographic examination showed dilated small intestines in 69.6% of the cows with TSC considering the distal location of the ileus. However, this was much lower compared with cows with small intestinal volvulus (87.1%) [38], small intestinal intussusception (96.0%) [17] and small intestinal strangulation (100%) [19]. By comparison, the caecum was not dilated in cows with small intestinal ileus, but it was in all cows with caecal torsion [24], in 24.0% of cows with TSC and in 13.5% of the cows with mesenteric torsion [18]. The spiral colon was dilated (an intestinal loop with a maximum diameter exceeding 5.0 cm; normal, 2.1 to 5.0 cm) [45] in 54.5% of cows with caecal dilatation [24]. Spiral colon dilatation occurred in 44.0 and 32.4% of cows with TSC and mesenteric torsion [18], respectively. Thus, dilatation of the caecum and spiral colon could signal caecal dilatation, mesenteric torsion or TSC but not small intestinal ileus. The differential diagnosis of TSC therefore includes mesenteric torsion and caecal dilatation (possibly accompanied by torsion and/or retroflexion). These three conditions also have close clinical similarities and are not easily differentiated. Cows with mesenteric torsion and TSC are usually more severely ill and have a more acute course of illness than cows with caecal dilatation. In addition, transrectal palpation of cows with mesenteric torsion often shows dilated small intestines along with dilated large intestines. However, a definitive diagnosis is often difficult. For these reasons, we recommend that cows with moderately to severely abnormal general condition, tachycardia, reduced or absent faecal output and palpable dilated small and large intestines undergo expeditious laparotomy. Medical treatment alone including the use of cholinergic drugs for cows with caecal dilatation [44] is contraindicated and could be fatal in cows with TSC. Of the cows in the present study, 65.5% had received medical treatment before referral resulting in a delay in surgical treatment and presumably accounting for the relatively low rate (44.8%) of discharged cows. Only cows with mesenteric torsion had a lower discharge rate (23.0%) [18], whereas the rate in cows with small intestinal intussusception (44.4%) [17] was similar to that of cows with TSC. Better outcomes were reported in cows with internal herniation (55.6%) [46], small intestinal strangulation (81.7%) [19] and ileal impaction (100%) [26]. In cows with caecal dilatation and severe acute illness, the main rule-out is TSC. A dilated spiral colon, detected clinically or ultrasonographically, further supports the diagnosis.

The question arises as to whether the cows in the present study had anatomical changes similar to those seen with heterotopy of the spiral colon [4, 9, 12]. Based on the surgical findings, complete ectopy of the spiral colon can be ruled out, but not mild (grade 1) or even moderate (grade 2) anomalies [4]; possible grade-1 or grade-2 changes may have been overlooked because of the severity of postmortem changes in the intestinal tract. Nevertheless, in cows with TSC, close attention should be paid to anatomical changes in the large colon.

## Conclusions

Dilated spiral colon and caecum, colic and reduced faecal output accompanied by an acute illness with tachycardia are characteristic of TSC. The main rule-outs are mesenteric and caecal torsion. Expeditious surgical treatment is crucial in cows with TSC.

### Electronic supplementary material

Below is the link to the electronic supplementary material.

Supplementary Material 1

Supplementary Material 2

## Data Availability

The datasets used and analysed for this study are available from the corresponding author on reasonable request.
