# Supplementary material for: Torsion of the spiral colon in cattle– a retrospective analysis of 58 cases
Source: Acta Vet Scand. 2024 Apr 15;66:17. doi: 10.1186/s13028-024-00738-w (PMC11017544; doi:10.1186/s13028-024-00738-w)
Supplement: Supplementary file 2 — Supplementary Material 2 [file 13028_2024_738_MOESM2_ESM.docx]

**Additional file 2**

**Laboratory findings in cattle with torsion of the spiral colon (means, medians, standard deviations, 95% CI, frequency distributions)**

| **Variable** |  | **Number** | **Per-** |
| --- | --- | --- | --- |
| **(Mean±sd, Median, 95% CI)** | **Finding** | **of cows** | **cent** |
| Haematocrit (n=58)  (median=37.0%, 95% CI=36.0-39.0%) | Normal (30-35%)  Decreased (28-29%)  Increased (36-56%) | 16  5  37 | 27.6  8.6  63.8 |
| Total leukocyte count (n=58)  (median=10,200 (/µL, 95% CI=8,800-12,400/µL) | Normal (5,000-10,000/µL)  Decreased (2,900-4,999/µL)  Increased (10,001-27,300/µL) | 25  3  30 | 43.1  5.2  51.7 |
| Total protein (n=57)  (mean±sd=77.1/±9.5 g/L, 95% CI=74.5-79.6 g/L) | Normal (60-80 g/L)  Increased (81-110 g/L) | 38  19 | 66.7  33.3 |
| Fibrinogen (n=57)  (median=5.0 g/L, 95% CI=5.0-6.0 g/L) | Normal (4-7 g/L)  Decreased (1-3.9 g/L)  Increased (7.1-11 g/L) | 38  8  11 | 66.7  14.0  19.3 |
| Urea (n=58)  (median=6.8 mmol/L, 95% CI=6.1-8.0 mmol/L) | Normal (2.1-6.5 mmol/L)  Increased (6.6-22.1 mmol/L) | 27  31 | 46.6  53.4 |
| Bilirubin (n=57)  (median=5.2 µmol/L, 95% CI=3.5-6.3 µmol/L) | Normal (0.4-6.5 µmol/L)  Increased (6.6-14.2 µmol/L) | 38  19 | 66.7  33.3 |
| Calcium (n=24)  (median=2.09 mmol/L, 95% CI=1.90-2.25 mmol/L) | Normal (2.30-2.60 mmol/L)  Decreased (1.52-2.29 mmol/L)  Increased (2.61-3.34 mmol/L) | 6  17  1 | 25.0  70.8  4.2 |
| Magnesium (n=24)  (median=1.16 mmol/L, 95% CI=1.04-1.43 mmol/L) | Normal (0.80-1.00 mmol/L)  Increased (1.01-1.94 mmol/L) | 7  17 | 29.2  70.8 |
| Inorganic phosphate (n=24)  (mean±sd =1.58±0.69 mmol/L, 95% CI=1.29-1.87) | Normal (1.30-2.40 mmol/L)  Decreased (0.58-1.29 mmol/L)  Increased (2.41-3.22 mmol/L) | 14  8  2 | 58.4  33.3  8.3 |
| Chloride (n=58)  (mean±sd=99.1±9.28 mmol/L, 95% CI=96.7-101.6 mmol/L) | Normal (96-105 mmol/L)  Decreased (74-95 mmol/L)  Increased (106-118 mmol/L) | 30  15  13 | 51.7  25.9  22.4 |
| Potassium (n=58)  (median=4.0 mmol/L. 95% CI=3.8-4.1 mmol/L) | Normal (4.0-5.0 mmol/L)  Decreased (2.3-3.9 mmol/L)  Increased (5.1-7.7 mmol/L) | 20  32  6 | 34.5  55.2  10.3 |
| AST (n=58)  (median=98.0 U/L, 95% CI=94.0-113 U/L) | Normal (42-103 U/L)  Increased (104-430 U/L) | 34  24 | 58.6  41.4 |
| γ-GT (n=58)  (median=20.0 U/L, 95% CI=18-21 U/L) | Normal (8-30 U/L)  Increased (31-154 U/L) | 55  3 | 94.8  5.2 |
| pH (n=39)  (mean±sd =7.37±0.06, 95% CI=7.35-7.39) | Normal (7.41-7.45)  Decreased (7.25-7.40)  Increased (7.41-7.48) | 10  26  3 | 25.6  66.7  7.7 |
| pCO_2_ (n=38)  (mean±sd =43.8±6.40 mmHg, 95% CI=41.7-45.9 mmHg) | Normal (35.0-45.0 mmHg)  Decreased (31.2-34.9 mmHg)  Increased (45.1-63.9 mmHg) | 23  2  13 | 60.5  5.3  34.2 |
| Bicarbonate (n=37)  (mean±sd=24.7±4.23 mmol/L, 95% CI=23.3-26.1 mmol/L) | Normal (20.0-30.0 mmol/L)  Decreased (15.2-19.9 mmol/L)  Increased (30.1-31.9 mmol/L) | 27  7  3 | 73.0  18.9  8.1 |
| Base excess (n=39)  (mean±sd=0.52±4.53 mmol/L, 95% CI=-0.95- +1.98 mmol/L) | Normal (-2 - +2 mmol/L)  Decreased (-10.1 - -2.1 mmol/L)  Increased (+2.1 - +8.9 mmol/L) | 11  10  18 | 28.2  25.6  46.2 |
| Rumen chloride (n=44)  (median=20.0 mmol/L, 95% CI=18.0-25.0 mmol/L) | Normal (≤ 30 mmol/L)  Increased (31-52 mmol/L) | 40  4 | 90.9  9.1 |
